# Olfactory Deficits in an Alpha-Synuclein Fly Model of Parkinson’s Disease

**DOI:** 10.1371/journal.pone.0097758

**Published:** 2014-05-30

**Authors:** Alex Y. Chen, Shouzhen Xia, Paul Wilburn, Tim Tully

**Affiliations:** 1 Dart Neuroscience LLC, San Diego, California, United States of America; 2 Cold Spring Harbor Laboratory, Cold Spring Harbor, New York, United States of America; 3 Graduate Program in Neuroscience, SUNY Stony Brook, Stony Brook, New York, United States of America; 4 W.K. Kellogg Biological Station, Michigan State University, Hickory Corners, Michigan, United States of America; Brigham and Women’s Hospital, Harvard Medical School, United States of America

## Abstract

Parkinson’s disease (PD) is the most common motor neurodegenerative disorder. Olfactory dysfunction is a prevalent feature of PD. It often precedes motor symptoms by several years and is used in assisting PD diagnosis. However, the cellular and molecular bases of olfactory dysfunction in PD are not known. The fruit fly *Drosophila melanogaster,* expressing human alpha-synuclein protein or its mutant, A30P, captures several hallmarks of PD and has been successfully used to model PD in numerous studies. First, we report olfactory deficits in fly expressing A30P (A30P), showing deficits in two out of three olfactory modalities, tested – olfactory acuity and odor discrimination. The remaining third modality is odor identification/naming. Second, oxidative stress is an important environmental risk factor of PD. We show that oxidative stress exacerbated the two affected olfactory modalities in younger A30P flies. Third, different olfactory receptor neurons are activated differentially by different odors in flies. In a separate experiment, we show that the odor discrimination deficit in A30P flies is general and not restricted to a specific class of chemical structure. Lastly, by restricting A30P expression to dopamine, serotonin or olfactory receptor neurons, we show that A30P expression in dopamine neurons is necessary for development of both acuity and discrimination deficits, while serotonin and olfactory receptor neurons appeared not involved. Our data demonstrate olfactory deficits in a synuclein fly PD model for exploring olfactory pathology and physiology, and for monitoring PD progression and treatment.

## Introduction

Parkinson’s disease (PD) is a motor degenerative disease, preferentially affecting the dopamine system. It is characterized by cardinal symptoms of bradykinesia, rigidity, tremor and postural instability. Olfactory impairments in PD were first documented in 1975 [1] and were considered the earliest symptom in the premotor phase of PD, Braak stage 1 [2]. The olfactory impairments can precede the appearance of motor symptoms by years [3]. Indeed, Lewy bodies (LBs) appear first in the olfactory bulb, before spreading to nuclei in brain stem, in amygdala (Stage 2–3) and then spreading into substantia nigra and other regions of the midbrain (stage 4: clinical disease stage), affecting motor functions.

Olfactory impairments in PD patients are prevalent, affecting 80 to 90% of both idiopathic PD patients and familial parkinsonism [4]–[6]. They comprise deficits in all three functional domains: odor threshold (acuity), identification and discrimination [7]–[9]. These deficits were shown to predate the onset of motor symptoms by years, particularly for relatives of patients diagnosed with PD [3], [10], [11]. Therapeutic intervention is best to be administrated early during disease progress, prior to significant neurodegeneration. For this reason, early diagnosis and differential diagnosis of PD from other motor disorders, such as vascular parkinsonism, Methylphenyltetrahydropyridine-induced parkinsonism, progressive supranuclear palsy (PSP) and corticobasal degeneration (CBD), are important and can be facilitated by assessing olfactory functions [9], [12]–[14].

Sporadic cases of PD predominate over heritable ones, and environmental factors such as oxidative stress appear to be involved [15]–[17]. Indeed, chronic administration of the herbicide paraquat (PQ) alone causes selective loss of nigral dopamine neurons with a concomitant emergence of motor deficits [18]–[20] and is used as a toxic PD model.

High expression of human α-synuclein (αSyn) directly contributes to PD pathology. Triplication, duplication and point mutations in αSyn are associated with dominantly inherited PD [21], [22]. Allele of synuclein A30P (A30P) is linked to early onset familiar PD. Together with other PD-associated genes, αSyn and A30P transgenic flies were made to model Parkinson’s disease [23]. When A30P is expressed in all neurons, flies show classic hallmarks of PD: the formation of Lewy body-like inclusions in aged animals, progressive and selective loss of dopaminergic neurons and progressive development of motor deficits, which is responsive to L-DOPA treatment [24]–[28].

In the present study, we examined the motor function in a pre-established A30P fly Parkinson’s disease model. Using A30P flies, we asked whether A30P synuclein flies would develop olfactory impairments. Then, we examined the chronological relationship between the occurrences of olfactory and motor impairments. Separately, we determined whether the olfactory modality of order acuity (OA), odor discrimination (OD) or both, were affected in A30P flies, using T-maze assays [29]. Then, we investigated whether the functions of OA and OD were sensitive to oxidative stress, and whether the OD impairment was odor-specific or non-specific.

## Results

### Aged A30P Flies Show Deficit of Climbing

**Figure 1:**
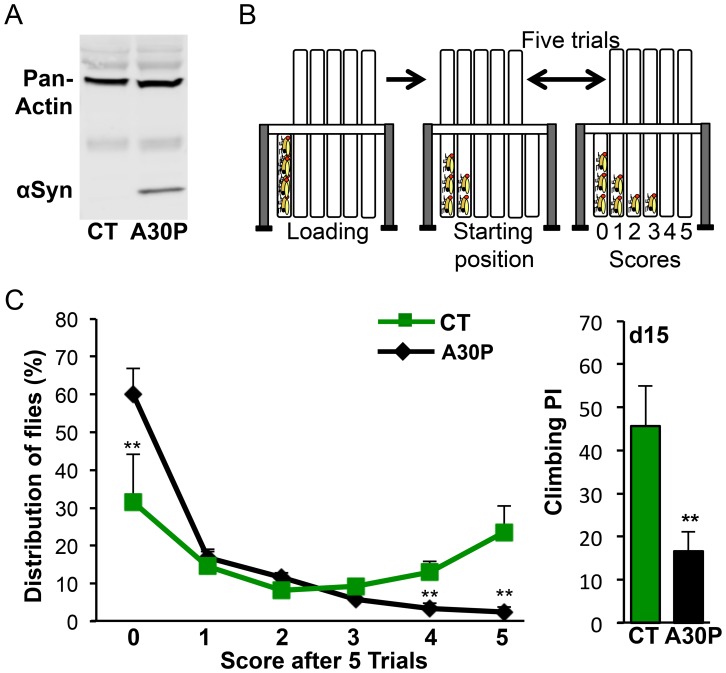
A30P flies show climbing deficit. (A) A30P expression in A30P flies. A30P protein from heads of five days old flies was probed using human-specific αSyn antibody. Pan-actin expression was used as an internal control. (B) Climbing assay. Climbing performance was measured based on negative geotaxis using countercurrent apparatus. By moving the upper five climbing tubes back and forward between each trial per test, flies would distribute between tubes 0 through 5. Each trial is 10 seconds. Details are in materials and methods. (C) Left: Fly distribution based on climbing performance. More CT flies reached the No.5 tube, while more A30P flies stayed in the No.1 tube. The percentage distribution (%) for a tube is [(# of flies in the tube)/(# of flies per test)]×100. The assays were performed with flies of fifteen days old. Fifty to eighty flies were used per trial. Ct *vs.* A30P: t_0_ (8) = 3.588, **P_0_ = 0.007; t_4_(8) = 3.770, **P_4_ = 0.0054; t_5_(8) = 3.846, **P_5_ = 0.0049. Right: The compounded climbing performance index (PI) from the left. PI = 100%×[Σ^5^_i_ = (# of flies)_i_×i/(# of flies per test)×5]. t(8) = 4.768, **P = 0.0014; CT is +/+: A30P/+; A30P is Elav/+: A30P/+, hereafter unless noted otherwise. Student *t*-test.

To verify A30P protein expression in a pre-established A30P PD fly model, we used immunoblotting and showed that A30P protein was expressed in the heads of A30P flies, carrying both a pan-neural Elav-gal4 driver and an UAS-A30P transgene, but not in Control (CT) flies that carried only UAS-A30P transgenes (Figure 1A). To verify reported motor deficit in A30P flies [25], we next examined the climbing performance of A30P flies, using Countercurrent apparatus. Countercurrent apparatus allowed us to monitor the climbing ability through multiple climbing trials in a test (Figure 1B). Details on the climbing assay can be found in the Methods section. We found a lower percentage of A30P flies reaching the far right fifth and sixth tube, receiving a score of 4 and 5, respectively, while a higher percentage of A30P flies (59.98%) was retained in the first tube after five trials, receiving a score of zero (**P<0.01; Figure 1C, left). A compounded climbing performance index (PI) based on fly distribution between 6 tubes showed 64% decrease of PI in 15 days old A30P (**P = 0.0014, Figure 1C, right), validating that A30P flies exhibit impaired motor function.

### A30P Flies Show Age-accelerated Deficits in Odor Acuity and Odor Discrimination

**Figure 2:**
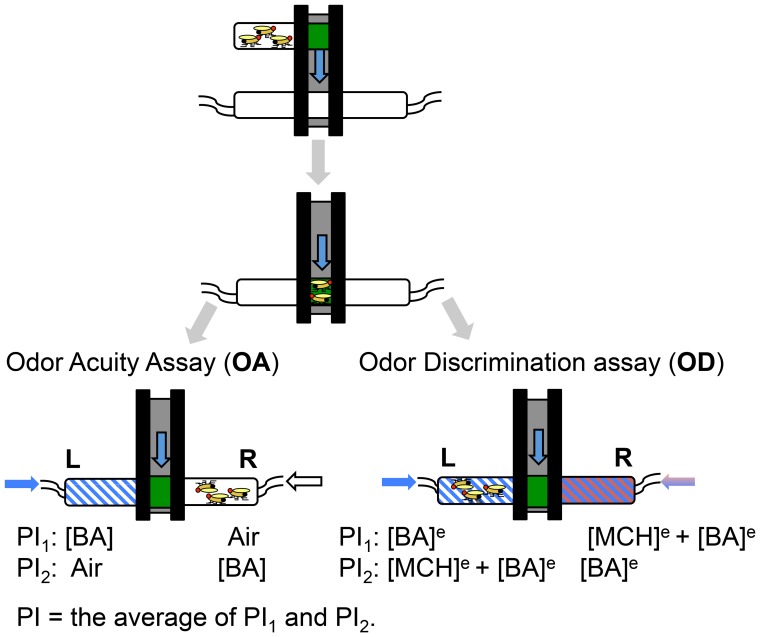
Fly odor acuity (OA) and odor discrimination (OD) assays. Fly odor acuity (OA) and odor discrimination (OD) were tested using T-maze apparatus. Flies loaded into T-maze were dropped to a choice point between [BA] and air in the odor acuity assay or between [BA]^e^ and [MCH]^e^+[BA]^e^ in the odor discrimination assay. To avoid an inherent directional reference in assays, an odor of a pair was delivered from one end of T-maze during the first trial (PI_1_) and from the opposite end during the second trial (PI_2_). A test comprised two trials. The performance index (PI) of a test is the average of PI_1_ and PI_2_. When the equilibrium concentrations of BA ([BA]^e^) and MCH ([MCH]^e^) were delivered from the opposite ends of T-maze, flies would show no preference for either [BA]^e^ or [MCH]^e^ by equally distributing between the two end-tubes. Flies with health odor discrimination would detect the presence of [BA]^e^, a foreground odor, in the background of [MCH]^ e^ and run to [MCH]^e^ tube, avoiding the [MCH]^e^+[BA]^e^ tube. The formula for PI calculation was expressed as a percentage of the absolute number of flies that were differentially distributed between two end-tubes, divided by the total number of flies in a trial. PI_1_ or _2_ = 100%×|(# of L)- (# of R)|/(# of total flies in a test).

We asked whether A30P expression would affect olfactory function in *Drosophila*. Olfactory functions comprised three major domains: odor acuity, odor discrimination and odor identification. Odor acuity (OA) and odor discrimination (OD) assays were performed using fly T-maze [29], allowing flies to make a spontaneous choice between an odor versus air in OA assay or between a mix of foreground and background odors versus a background odor alone in the OD assay (Figure 2) [30]. Detailed description of both assays can be found in figure legend and the methods section.

**Figure 3:**
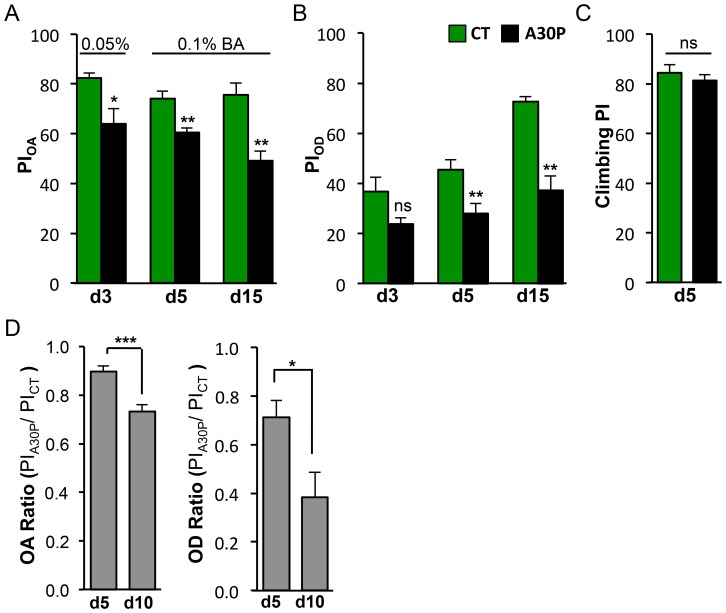
A30P flies show age-accelerated deficit in odor acuity and odor discrimination. (A) A30P flies showed decreased odor acuity (d3: t(6) = 2.916, *P = 0.0268; d5: t(10) = 3.982, **P = 0.0026; d15: t(5) = 4.046, **P = 0.0099). Odors used are 0.05% BA *vs.* air on day3 and 0.1% BA versus air on day5 and day 10. (B) Aged A30P flies showed decreased performance in odor discrimination (d3: t(8) = 2.094, *P = 0.0695; d5: t(14) = 3.058, **P = 0.0085; d15: t(6) = 5.915, **P = 0.0010). Odor options were 1.5% BA made in 15% MCH background and 15% MCH. (C) A30P flies showed normal climbing on day 5 (t(14) = 0.7625, *P* = 0.480). (A–C) Olfactory deficits preceded motor deficit. (D) Aging exacerbated both OA and OD deficits in A30P flies. The PIs for OA and OD were presented as ratios of PI_A30P_ over PI_CT_. Odors used in OA assays were 0.1% BA and air. Odors used in OD assays were the same as in Figure 2B. OA ratio: t(14) = 4.563, ***P = 0.0004; OD ratio: t(7) = 2.680, *P = 0.0316, Student *t*-test.

Olfaction is a sensory function that declines with age [31]–[34]. In OA assay, we measured flies’ ability to detect sub-threshold concentrations of benzaldehyde (BA). We found A30P flies as young as three-days old showed olfactory impairment in detecting 0.05% BA, and such impairment progressed to inability to sense 0.1% BA on day five and fifteen (Figure 3A).

In OD assay, we measured flies’ ability to discriminate between 1.5% BA from a background odor of 15% of 4-methyl-cyclohexanol (MCH). The concentrations of both odors used in OD assay were at a bioequivalent concentrations, meaning that while 1.5% BA and 15% MCH were presented at the opposite ended of a T-maze, flies would show equal preference for 1.5% BA and 15% MCH, by distributing 50∶50 at the two T-maze ends [30]. Also, 15% of MCH was pre-determined to induce a saturated behavior response, meaning that higher than 15% of MCH would not produce a higher score [30]. As a result, flies that failed to discriminate BA from MCH would not produce a higher behavior score by misrecognizing BA+15% MCH side containing higher than 15% of MCH. The odor discrimination function appears to initially improve with age in control animals (CT) and starts to show impairment on day five in A30P flies (Figure 3B). Both OA and OD impairments in A30P flies manifested on day five, when the animals still retained normal motor function, suggesting olfactory deficits preceded motor function decline (Figure 3A–C).

Age-dependent pathologies are a key feature of degenerative diseases. To determine whether aging would exacerbate OA and OD deficits, we calculated the PI_A30P_/PI_CT_ ratios in 5 and 10 day old flies. The OA and OD ratios significantly decreased on day 10, compared with day 5. OA decreased 16.31% (***P = 0.0004), and OD decreased 23.25% (*P = 0.024; Figure 3D), suggesting aging worsened both olfactory deficits.

### Odor Acuity and Discrimination are Sensitive to Oxidative Stress

**Figure 4:**
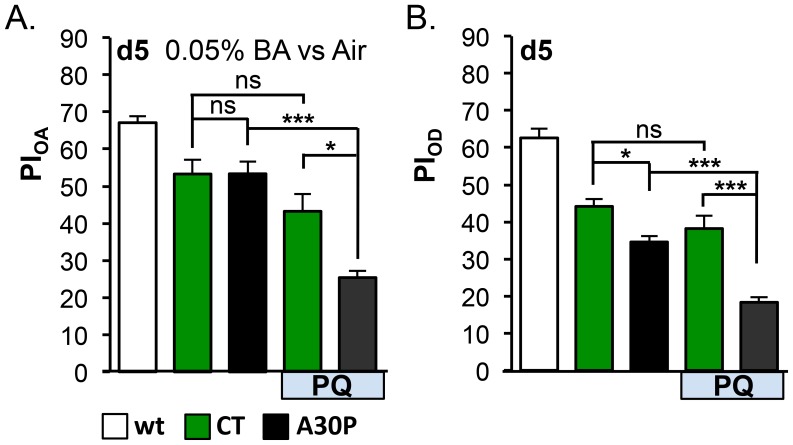
Oxidative stress exacerbated olfactory deficits in A30P flies. (A) Odor acuity (OA) and (B) odor discrimination (OD) assays. Five-days old A30P flies showed normal olfactory acuity (0.05% BA vs. air) and mild discrimination deficit. Paraquat (PQ) feeding enhanced OA and OD deficits in A30P (For CT+PQ *vs.* A30P+PQ comparison: F_OA_1,12 = 6.243, *P = 0.0280; F_OD_1,23 = 5.5152, *P = 0.0329). In OA assays, the mean differences of CT *vs.* CT+PQ was 10.04% (n.s.: *P*>0.05) and of A30P *vs.* A30P+PQ was 27.96% (***P<0.001). In OD assays, the mean differences of CT *vs.* CT+PQ was 5.97% (n.s.: *P*>0.05) and of A30P *vs.* A30P+PQ was 16.25% (***P<0.001). A30P and PQ-feeding were variables significantly interacting with each other (Interaction: *P_OA_ = 0.028, *P_OD_ = 0.033), suggesting an enhancement effect. PQ was 5 mM; wt was an internal control for apparatus assays. Odors used in OA were 0.05%BA and air. Odors used in OD assays were the same as in Figure 3B. Other comparisons: ***P<0.001, **P<0.01, *P<0.05, ns: non significant. Two-way ANOVA followed by Tukey Post-hoc tests among CTs and A30Ps with or without PQ-feeding.

Oxidative stress has been linked to Parkinson’s disease by both post-mortem pathological [35]–[37] and by epidemiological studies [38], [39]. Paraquat (PQ: N,N′-dimethyl-4,4′-bipyridinium dichloride) is one of the most widely used herbicides, and was shown to induce selective dopamine cell death in substantial nigra when systematically administrated in rodent PD models [18], [19], [40]. We asked whether olfactory acuity and discrimination of A30P flies could be affected by oxidative stress. To do so, we fed newly enclosed adult flies with 5 mM PQ mixed into food for the duration of the experiment, and tested OA and OD performances in the animals. Five day old A30P flies showed healthy OA function in detecting 0.05% BA and mild OD impairment (*P = 0.0329). The 0.05% BA used in the PQ OA assay, which was lower than what was used in the OA assay on day5 in Figure 3A, is more challenging. In the PQ OD assay, the same odor pair and concentrations of odors as in Figure 3B were used. We found PQ feeding significantly enhanced both OA and OD deficits in A30P, compared to CT (***P<0.001, Figure 4 A and B), suggesting oxidative stress worsened both olfactory deficits.

### Aged A30P Flies Exhibited Non-odor-specific Discrimination Deficit

*Drosophila* has a diverse family of odorant receptor (Or) genes. Expression of each Or is restricted to a subset of the olfactory receptor neurons (ORNs) that project to the same glomerulus in the antennal lobe, a mammalian olfactory bulb analog, where odor information is first encoded [41], [42]. Studies show that quality, quantity and duration of odor exposure activate ORNs in a specific manner. Aromatic odors often activate fewer ORNs, compared to most alcohols, which activate a broader range of ORNs [43].

**Figure 5:**
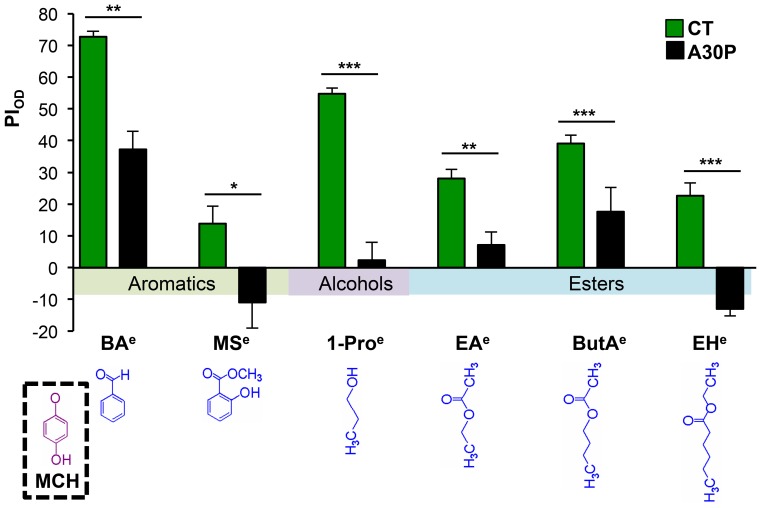
A30P flies showed non-odor-specific discrimination deficits. Fifteen-days old flies were tested for the ability to discriminate the presence of different odors made in 15% MCH versus 15% MCH alone. Chemical structures of each odor are shown and arranged from aromatics on the left, to esters on the right, based on structure similarity to MCH, the background odor. Behavior-equivalent concentrations used were 1.5% of BA^e^, 20% MS^e^, 1% of 1-Pro^e^, 20% of EA^e^, 10% of ButA^e^ and 5% of EH^e^ made in 15% of MCH^e^. BA: benzaldehyde, MS: methyl salicylate, 1-Pro: 1-propanol, EA: ethyl acetate, ButA: butyl acetate, EH: ethyl hexanoate. For CT vs. A30P comparisons from left to right, t_BA_(6) = 5.915,**P = 0.001, t_MS_(7) = 2.,*P = , t_1-Pro_(6) = 8.986, ***P = 0.0001, t_EA_(8) = 3.651,**P = 0.0065, t_ButA_(12) = 4.750,***P = 0.0005, and t_EH_(11) = 8.404,***P<0.0001; Student *t*-test.

We asked whether OD deficit seen in A30P fly is restricted to its ability to discriminate a particular class of odor chemicals. Odor chemical class specificity would suggest that OD deficit in A30P flies affects a specific subset of Or-expressing neurons. We chose a panel of five odors: BA, Methyl Salicylate (MS), 1-Propanol (1-Pro), ethyl acetate (EA), Butyl acetate (ButA), and ethyl hexanoate (EH) to represent three classes of chemical structures: aromatics, alcohol, or esters. Together, the five odorants can activate or inhibit 23 out of the 24 types of functional Or-expressing neurons [43]. We found fifteen day old A30P flies showed non-odor specific discrimination deficit (Figure 5), suggesting that deficit in the odor discrimination is not likely mediated by impairment of any particular ORN type.

### Odor Acuity and Discrimination Deficits in A30P Aged Flies are Dopaminergic

Dopamine neurons are selectively more susceptible to the toxicity of synuclein and A30P overexpression [44], [45]. We asked whether OA and OD deficits in A30P flies were due to: (a) functional impairments of dopamine neurons in olfaction transmission, and/or (b) functional impairments of ORNs. To answer (a), we restricted A30P expression to dopamine neurons, using tyrosine hydroxylase (TH)-gal 4 driver. TH is an enzyme required to convert L-tyrosine into L-DOPA, a precursor to making dopamine. In comparison, we restricted A30P expression to cholinergic neurons, using Cha (choline acetyltransferase)-gal4 driver. Cha is an enzyme required to add acetyl-CoA to choline to produce neurotransmitter acetylcholine. To answer (b), we restricted A30P expression in ORNs, using Or83b-gal4 driver. Or83b is an olfactory co-receptor, broadly expressed in all ORNs to mediate responses to all odors [46]. We measured PI_OA_ and PI_OD_ of fifteen day old A30P and CT flies.

**Figure 6:**
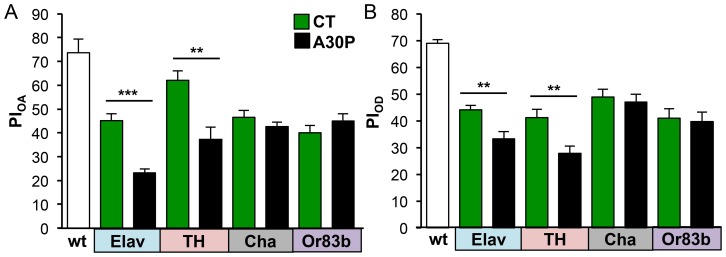
A30P expression in dopaminergic neurons causes odor acuity and discrimination deficits in aged A30P flies. Fifteen-days old A30P flies expressing A30P under Elav-gal4 (Elav), TH-gal4 (TH), Cha-gal4 (Cha) or Or83b-gal4 (Or83b) drivers were tested for odor acuity (A) and the performance of odor discrimination (B). Young wild-type flies of 1–2 days old were used as internal assay control. Only A30P expressed in dopamine neurons, A30P_TH_, showed olfactory acuity and discrimination deficits as seen in A30PElav. The corresponding controls for each comparison were CT_Elav_ (Elav/+; +/+; +/+), CT_TH_ (+/+; +/+; TH/+), CT_Cha_ (+/+; +/+; Cha/+), and CT_Or83b_ (Or83b/+; +/+; +/+). OA: t_Elav_(14) = 6.388, ***P<0.0001, t_TH_(14) = 3.773, **P = 0.0021; OD: t_Elav_(14) = 3.338, **P = 0.0049, t_TH_(14) = 3.257, **P = 0.0057; Student *t*-test.

A30P flies showed OA and OD deficits with Elav-driven A30P in all neurons (OA_Elav_: ***P = 1.69×10^−5^; OD_Elav_: **P = 0.005) and with TH-driven A30P in only dopamine neurons (OA_TH_: **P = 0.002; OD_TH_: **P = 0.006). In contrast, Cha- and Or83b-driven A30P expression in cholinergic and ORNs, respectively, did not show OA and OD impairments, compared to CT (OA_cha_: *P = *0.398; OD_cha_: *P = *0.700; OA_Or83b_: *P = *0.200; OD_Or83b_: *P = *0.773; Figure 6 A and B). These results suggested that dopamine neurons were the targets of A30P protein toxicity, affecting OA and OD performances as seen in Elav-A30P flies.

## Discussion

Olfactory deficits and misregulation of synuclein expression are prevalent in Parkinson’s and Alzheimer’s patients. Prior to our study, olfactory deficits in a synuclein PD fly model were not known. Here, through examining olfactory acuity and odor discrimination, we report impairments in both olfactory domains in Elav-A30P flies that precede appearance of motor dysfunction and are accelerated by oxidative stress with paraquat feeding. Presented with different odor pairs, A30P flies show impairments in odor discrimination in non odor specific manner, as reported in PD human patients [47]. Limiting A30P protein expression to dopamine neurons caused impairments in odor acuity and discrimination similar to those in flies with Elav-driven A30P in all neurons, suggesting impairments are mediate by dysfunction in dopamine neurons. Consistent with our observations, mice expressing wild-type human synuclein, driven by Thy1 promoter, show deficits in both odor acuity and discrimination, tested by buried pellet and block assays [48]. Odor acuity was also impaired in transgenic rats, expressing both A30P and A53T synuclein under TH promoter, prior to appearance of motor symptoms [49], suggesting a direct involvement of dopamine neurons, as this study also indicated.

Olfactory organization is conserved between mammals and insects: axons of olfactory receptor (sensory) neurons are wired and converged based on the type of odorant receptor (Or) expressed. Projection axons are organized into distinctive spherical neuropils, or glomeruli, in the antennal lobe (AL) in *Drosophila* or the olfactory blub (OB) in mammals, respectively [50].

Whether deterioration of olfactory function is associated with central dopamine deficiency is continuously debated. Olfactory dysfunction in PD patients was thought to plateau without further deterioration to reflect disease progression [47]. However, careful sample selection, closer follow-up and an improved mechanistic understanding have been proposed to reveal the progression olfactory impairments with disease advancement [51]. Common complications are an aging-dependent natural decline in olfactory function and compensatory responses. A30P PD flies provide an excellent model to study the dynamics of olfactory deficits and other PD-related pathologies. Compared to mouse and rat models, flies have a much shorter lifespan and faster disease progression. Olfactory deficits can be detected in as early as five day old adults before motor deficit becomes apparent in 15 day old flies. A fly PD model allows studies with large animal populations to test an array of odor concentrations for different age groups, needed in olfactory studies, and provides the necessary statistical power to draw conclusions.

Whether olfactory deficits in PD originate from sensory, central or motor neuron deficits is not known and is difficult to disentangle in human or mouse studies. Spontaneous, in contrast with conditioned and experience-dependent [52], odor detection and discrimination were proposed to be processed locally within the antennal lobe in insects [53], [54] and the olfactory bulb in mammals [55]–[60]. Results of OA and OD assays in this study depend on spontaneous responses and, thus, argue against involvement of the central nerve system. Known odor receptor neurons (ORNs) are mostly glutaminergic [61], [62] and acetylcholinergic (ACh) [63], [64]. At the same time, dopamine neurons were found to innervate the center of neurpiles and in the glial layer at intraglomeruli [65], indirectly modulating the outputs of olfactory sensory neurons in flies. Therefore, normal OA and OD function of Or83b-A30P flies and olfactory deficits seen in TH-A30P flies together suggest that olfactory deficits in A30P flies were not due to dysfunction of ORNs but due to local dopamine neurons modulating ORNs in the antennal lobe. A direct involvement of local antenna lobe neurons in olfactory perception was also supported by a recently study, which showed reduced neural activity in electroantennogram and reduced marker for neuronal active zones in PINK null flies [66] - a transgenic fly with autosomal recessive PD.

Deficit in motor neurons was proposed to contribute olfactory impairments in PD patients. PD patients showed significantly decreased sniff airflow and volume. When asked to sniff vigorously, patients’ performances in odor acuity and identification improved [67]. Indeed, active sensing by sniffing (inhaling air into the nasal cavity) is the first step and a requirement for normal olfactory function for human and rodents. It is therefore difficult to disassociate the prime cause of olfactory deficits between the dysfunctions of motor or sensory neurons. On the contrary, flies sample odorants in air directly through odor sensory neurons that reside in the sensillum, and do not require motor function, such as sniffing. Therefore, olfactory deficits in A30P flies support direct involvement of sensory impairment, specifically, mis-modulation of sensory neuron output by A30P expression in dopamine neurons. This is also consistent with observations that increased sniffing effort in PD patients does not restore olfactory function back to normal and is only beneficial to the worst performers that tend to show greatest motor dysfunction [67].

Constitutive neurogenesis in OB is an active event, where activity is highly responsive to injuries. Inhibition of neurogenesis in OB leads to olfactory impairments [68]. Both increase and decrease of neurogenesis in olfactory bulbs were reported Parkinson’s patients and PD animal models [49], [69]–[73]. The discrepancies may be due to the time points when neurogenesis was examined: early or late during PD progression. Possible compensation mechanism that leads to upregulated neurogenesis was proposed to explain the increased neurogenesis in OB in PD patients. In transgenic PD models, the discrepancies may be also due to methods of expressing synculein or its mutant genes: acute expression by using a conditional system or constitutive expression by using a promoter. The kinetics of synuclein protein expression may affect regulation of neurogenesis. In flies, one prominent site of adult neurogenesis in *Drosophila* brain is at the conjunction between antennal lobes and antennal nerve bundle [74]. Extensive genetic tools available in flies allow to resolve discrepancies and to investigate the role of neurogenesis in PD pathology and in olfactory function to determine whether altered neurogenesis is a primary or secondary effect of olfaction impairments in s synuclein fly model. By comparing conditional and continuous methods of expressing synuclein in the same genetic background, *Drosophila* PD model can help delineate the effect of synuclein on neurogenesis.

## Materials and Methods

### Flies

We used wild-type 2U flies, a pan-neural Elav-gal4 (Elav) promoter, dopaminergic specific Th-gal4 (TH) promoter, olfactory-neuron Or83b-gal4 (Or83b) promoter, cholinergic neuron Cha-gal4 (Cha) promoter from Bloomington Fly center, and a UAS-human αSyn A30P (A30P) from Mel B. Feany Lab. All flies were outcrossed to 2U for six or more generations to equilibrate genetic background before experiments. Flies were raised under a 12∶12 light:dark cycle at 25°C and 70% humidity incubator. PQ was administrated by rearing adult flies in vials with regular food [75] mixed with PQ. PQ food was made by completely melting the regular fly food and allowing it to cool at 57° before adding PQ. PQ-containing fly food was stored up to ten days at 4°C before usage.

### Climbing Assay

Startle-induced negative geotactic climbing was used to measure climbing performance with a countercurrent apparatus [76]. During testing, a group of 20–30 flies was placed into the far left “bottom” tube at the loading position. Flies were gently tapped down to the bottom of tube three times with a force of about 1 to 1.5 kg at the frequency of one tap per second. The top “receiver” tubes were quickly moved to the left, allowing flies 10 seconds to climb up into the top receiver tube at a starting position in each trial. Flies that succeeded in climbing up to the receiver tube were transferred to the next bottom tube at the end of the 10 second period by moving the array of top tubes one slot to the right, followed by gentle tapping and prompt placement of top tubes back to the starting position. These steps repeated five times, providing flies five trials to climb into the top tube. At the end of the test, flies in the far right bottom tube that successfully climbed to the top receiver tube in all five trials received a score of 5. Flies that remained in the first bottom tube at the end of five trials failed to climb above a tube’s length in each trial and received a score of 0. A Performance index (PI) was calculated as shown in Figure 1 legend.

### Olfactory Assays

Flies were collected and kept in clean bottles for one day before testing. Groups of 50 to 80 flies were transferred into fresh food vials on the test day. All odors were made fresh in mineral oil using odor free glass vials. To eliminate odor and any asymmetric biases in the testing machines, one group of flies was tested with a given odor (or odor mixture) delivered into the left arm of the T-maze (PI_1_), and a second reciprocal group was tested with the same odor (or odor mixture) delivered into the right arm (PI_2_). The average of two performance indices (PIs) from these two groups of flies was defined as a score for n = 1. Two olfactory assays performed were odor acuity (OA) and odor discrimination (OD). In the odor acuity (OA) assay, different concentrations of benzaldehyde (BA) were paired with odor-free mineral oil (air). Normally, flies can detect the presence of BA at a very low concentration [77] and show preference towards or away from BA, depending on BA concentration. Flies with OA deficits exhibit a higher threshold of detecting the presence of BA odor and a shifted olfactory response to BA concentration. In odor discrimination (OD) assay, flies were tested for ability to detect the presence of a behavior-equivalent concentration of BA (BA^e^) odor in the background of methylcyclohexanol (MCH^e^) odor versus MCH^e^ odor alone. The behavior-equivalent concentrations of two odors are those that produce a PI score close to zero in flies presented with the two choices at the opposite arms of a T-maze [30]. A zero score means that flies would show no odor preference and distribute 50/50 in both arms. Normal flies can identify the presence of BA in the background of MCH and avoid the arm filled with MCH^e^ and BA^e^ odor mixture. However, flies with an OD deficit, would show difficulties in detecting BA^e^ in MCH^e^ background, resulting in a lower PI score, suggesting failure to discriminate the presence of BA^e^ in a strong MCH^e^ odorant background.

### Immunoblotting

Thirty fly heads were homogenized in 150 ul 2x Laemmli sample buffer (Bio-Rad, Hercules, CA) and centrifuged for 20 minutes at 13,000×g at 4°C. Protein extract from about five heads was used per lane on SDS PAGE gels for detecting and visualizing the expression transgene, human αSyn protein. Antibodies used were human specific anti-αSyn (1∶1000, Cell Signaling Technology, Inc., Danvers, MA) and a pan-actin antibody (1∶1000; Sigma-Aldrich Corp., St. Louis, MO).

### Statistics

Due to the nature of their mathematical derivation, PIs are distributed normally. Data were subjected to Student *t*-test, one-way or two-way ANOVA test, followed by Tukey post-hoc tests. All data were presented as “mean ± SEM”. All statistics were analyzed using Prism 6 (GraphPad Software, Inc.).
